# Mitochondrial transplantation and transfer: The promising method for diseases

**DOI:** 10.55730/1300-0152.2665

**Published:** 2023-10-18

**Authors:** Gökhan Burçin KUBAT

**Affiliations:** Department of Mitochondria and Cellular Research, Gülhane Health Sciences Institute, University of Health Sciences, Ankara, Turkiye

**Keywords:** Mitochondria, mitochondrial transplantation, mitochondrial transfer

## Abstract

Mitochondria are organelles that serve as the powerhouses for cellular bioenergetics in eukaryotic cells. It is responsible for mitochondrial adenosine triphosphate (ATP) generation, cell signaling and activity, calcium balance, cell survival, proliferation, apoptosis, and autophagy. Mitochondrial transplantation is a promising disease therapy that involves the recovery of mitochondrial dysfunction using isolated functioning mitochondria. The objective of the present article is to provide current knowledge on natural mitochondrial transfer processes, in vitro and in vivo applications of mitochondrial transplantation, clinical trials, and challenges associated with mitochondrial transplantation.

## 1. Introduction

As the “powerhouse” of the cell, mitochondria are found in the cytoplasm of cells and generate the majority of the energy as ATP. The diameter and length of mitochondria vary from 0.5 to 1 m and 0.5 to 10 m, respectively (Caicedo et al., 2017). The morphology of mitochondria differs considerably based on the cell type. For instance, fibroblast mitochondria tend to form long filaments, whereas hepatocyte mitochondria seem to be regularly spherical (Youle and van der Bliek, 2012).

Acute and chronic diseases can be triggered by a loss of function in the mitochondria which has become the major organelle in regulating cellular energy production. A disturbance in electron transport chain activity and the transfer of vital metabolites into mitochondria give rise to an impairment in mitochondrial function at the molecular level (Nicolson, 2014). Mitochondria-targeted antioxidants (SS31, Vitamin E, etc.), stem cell therapy, and exercise are all common in the treatment of mitochondrial dysfunction-related diseases (Akın et al., 2021; Kubat et al., 2023).

Mitochondrial transplantation is a promising therapy option for mitochondrial disorders. In human clinical studies, this form of therapy has been demonstrated to be effective in the treatment of cardiac ischemia after reperfusion (Emani et al., 2017; Guariento et al., 2021). It has also been found in preclinical research to be effective in treating mitochondrial dysfunction in the heart, kidney, liver, lungs, aging, toxicity, and brain (Moskowitzova et al., 2020; Gokhan Burcin Kubat et al., 2021; Ulger et al., 2021; Alemany et al., 2023; Javani et al., 2023; Ulger and Kubat, 2023).

Differential or gradient centrifugation can be utilized for mitochondrial isolation methods (Gostimskaya and Galkin, 2010). A quick mitochondrial isolation process should be selected for therapeutic purposes. McCully et al. generated a quick and simple isolation procedure by applying several filters, however, mitochondria isolation could take longer using commercial kits (McCully et al., 2016; Wang et al., 2019).

Different approaches have reported success with the transplanting of mitochondria isolated from soleus, rectus abdominis, stem cells, and exogenous mitochondria have been demonstrated to be incorporated in vitro and in vivo applications by direct injection, systemic and intranasal administration, coincubation, and cell-mediated transfer across multiple studies (Ulger and Kubat, 2022). Autologous, allogeneic, and xenogeneic mitochondria can be utilized for injection (Kubat et al., 2021).

The objective of this article is to give a summary of the therapeutic advantages and basic mechanisms of mitochondrial transplantation and transfer. It also discusses the methods for in vitro and in vivo applications of mitochondrial transplantation and transfer, clinical trials, and challenges associated with mitochondrial transplantation.

## 2. Natural mitochondrial transfer

Mitochondria are extensively connected organelles that can perform ongoing fusion and fission as well as dynamic shape remodeling (Mishra and Chan, 2016). For a long time, mitochondria were believed to be localized to the cytoplasm, although they move across cells and regularly modify their programming (Rafelski, 2013). A recent study suggests that mitochondria would possibly migrate across cell borders and within mammalian cells due to their segregation within cells and their dynamic nature (Torralba et al., 2016).

Extracellular vesicles (EVs), Gap Junctions (GJCs), Tunneling Nanotubes (TNTs), and Extrusion are the most widely recognized molecular pathways enabling the intercellular movement of mitochondria (Figure).

TNTs provide for the interchange of organelle or cytoplasmic molecules (Torralba et al., 2016) (Figure A). Typically, TNTs only allow for one-way mitochondrial transfer from the cell that generated the TNT to the cell that is the receptor (Bukoreshtliev et al., 2009). They have a diameter of around 200 nm and a length of about 100–150 μm (Sahinbegovic et al., 2020). In 2004, Rustom et al. identified organelle migration across mammalian cells with TNTs, while Spees et al. reported natural mitochondrial transfer from mesenchymal stem cells (MSCs) to different cells in 2006 (Rustom et al., 2004; Spees et al., 2006). When cells are exposed to stress, p53 expression increases, and the AKT-phosphoinositide 3-kinase (PI3K)-mammalian target of rapamycin (mTOR) signaling pathway is activated, causing TNTs to form between cells and mediating transcellular transport of organelles such as mitochondria (Wang et al., 2011). MSCs promote survival and repair cell damage in an ischemia-reperfusion model by transferring their mitochondria to H9c2 cells via TNTs (Han et al., 2016). Mitochondrial transfer from MSCs minimizes lung damage (Li et al., 2014), and promotes the capacity of alveolar macrophages via TNTs (Jackson et al., 2016). TNT-mediated mitochondrial transfer from endothelium to cancer cells improves chemoresistance (Pasquier et al., 2013). MSCs provide mitochondria to myeloma cells through TNTs in multiple myeloma, increasing their proliferation (Boise and Shanmugam, 2019).

EVs serve as carriers for cell-to-cell communication (Mittelbrunn and Sánchez-Madrid, 2012) (Figure B) and following biogenesis and nano diameter, they are divided into three broad classes including exosomes, microvesicles, and apoptotic bodies (Meng et al., 2020). EVs containing mitochondria have also been found to be released by cells via an EV-cell fusion event (Torralba et al., 2016). As shown in astrocytes and mesenchymal stem cells, larger EVs can include mitochondrial particles including mitochondrial DNA (mtDNA) (Phinney et al., 2015). Astrocytes generated mitochondrial materials that were partially absorbed by injured neurons for neuronal survival (Hayakawa et al., 2016). Myeloid-derived cells release microvesicles (MVs), which transfer mitochondria (Hough et al., 2018).

GJCs serve as direct communication pathways between two adjacent cells by hemichannels (Fiori et al., 2014) (Figure C). Cx43 hemichannels on mitochondria help regulate the equilibrium of mitochondrial calcium, but they may adversely impact mitochondria and trigger apoptosis (Gadicherla et al., 2017). Cx43 participates in mitochondrial transcellular movement. Alveolar cells with CX43-high expression sites are colonized by bone marrow mesenchymal stem cells (BMSCs) (Islam et al., 2012) GJCs facilitate the mitochondrial transfer from BMSCs to neurons (Li et al., 2019).

Another potential process of mitochondrial transfer across cells is mitochondrial extrusion (Ulger and Kubat, 2022) (Figure D). Extrusion is a precise process that releases mitochondria or mitochondrial material from cells. The contents of the mitochondria, including oxidized mitochondrial nucleoids, can be extruded by neutrophils (Caielli et al., 2016).

The natural mitochondrial transfer appears to promote multifunctional cellular functioning as well as tissue regeneration in pathological situations of different physiological systems (Liu et al., 2021).

## 3. Artificial mitochondrial transplantation and transfer: In vitro applications

In regard to artificial mitochondrial transplantation and transfer, Clark and Shay et al. first revealed that mitochondrial transplantation could successfully transfer to mammalian cells via the in vitro coincubation method. The lack of success of simple coincubation to transfer mitochondria into cells demonstrates that this method is not effectively successful between different types of cells. The findings of Clark and Shay for mitochondrial transfer have paved the way for more developments in the area (Clark and Shay, 1982). Elliott et al. coincubated malignant MCF7, MDA-MB-231, and ADR-Res cells with mitochondria derived from immortalized breast epithelial cells. However, the original immortalized breast epithelial cells could not be entered by isolated mitochondria from the immortalized breast epithelial cells (Elliott et al., 2012). The coincubation is an effective method for investigating the different areas of mitochondrial transplantation because it is performed in multiple aspects of in vitro studies in current literature.

King and Attardi then developed the first mitochondrial transfer method by applying invasive equipment. Microinjection of chloramphenicol-resistant mitochondria into 143BTK and HT1080-6TG human cells was also performed utilizing a 1-micron needle (King and Attardi, 1988). Due to a limited number of transplanted cells and transplanted mitochondria per cell, this method proved less productive than the coincubation method.

Mitopunch was an innovative approach for mitochondrial transplantation and can generate recipient cells with distinctive mtDNA-nDNA combinations and completely transfer mitochondria to several different types of cells (Patananan et al., 2020).

Wu et al. designed a photothermal nanoblade for direct cytoplasmic delivery of various compounds into mammalian and the results of metabolomics suggest that integrating a nanoblade to modify the mtDNA haplotype in somatic mammalian cells is a controllable, reproducible, and general technique (Wu et al., 2016). There are challenges with the technological tools, the knowledge needs, and their applicability to very few cells.

The Mito-Ception method permits the separated mitochondria from MSCs to be transferred to glioblastoma stem cell lines (Nzigou Mombo et al., 2017). Caicedo et al. observed that mitochondria of MSCs transferred using Mito-Ception improved the function of MDA-MB-231 endogenous mitochondria and increased both energy metabolism and function of the targeted cells (Caicedo et al., 2015). Mito-ception, on the other hand, showed the most effective survival of transplanted mitochondria and an improved capacity to preserve the activity of mitochondrial respiration (Jain et al., 2023). Due to concerns like membrane rupture, this method could be a suitable way to compare the delivery of direct mitochondria into the cells.

The Magnetomitotransfer method includes anti-TOM22 magnetic beads and a magnetic plate. Macheiner et al. identified this method after inserting magnetic beads with attached mitochondria into donor cells (Macheiner et al., 2016). Mitochondria were transferred into cells more effectively using the magnetomitotransfer method. This transfer was also more quickly, which resulted in an elevated density of transplanted mitochondria. Notably, transplanted mitochondria dramatically increased cell respiration (Macheiner et al., 2016). Nonfunctional mitochondrial particles can also be bound by anti-TOM22, reducing the efficiency of the method.

A delivery mechanism that includes Pep-1 is a different method for mitochondrial transfer. Pep-1 serves as a pacemaker and it promotes cellular uptake for the entry of small organelles into the cell (Chang et al., 2017). Mitochondrial transplantation using Pep-1 enhanced mitochondrial resistance to 6-OHDA neurotoxicity and maintained cell survival (Chang et al., 2016). It is challenging to compare the findings due to the limited literature, variations in the in vitro methods, and the assessment of transfer efficiency.

## 4. Artificial mitochondrial transplantation and transfer: In vivo applications

Studies on mitochondrial transplantation have recently focused on cardiovascular, musculoskeletal, renal, liver, and neurological disorders. Direct tissue injection, systemic circulation-based delivery to injured tissue, and intranasal administration have been performed for in vivo applications of mitochondrial transplantation (Table).

In neurological disorders, there is an impairment in ATP synthesis, an increase in reactive oxygen species (ROS) stress, and a reduction in calcium homeostasis because energy metabolism is rendered dysfunctional due to maintaining mitochondrial degeneration (Guo et al., 2013). In the acute ischemia stroke model, Pourmohammadi-Bejarpasi et al. isolated mitochondria from human umbilical cord-derived mesenchymal stem cells (hUC-MSCs) and transplanted them to the intracerebroventricular area. They found that mitochondrial transplantation improved motor ability, decreased the effects of reperfusion/ischemia, eliminated apoptosis, attenuated microglia activation process, and eliminated infarction size (Pourmohammadi-Bejarpasi et al., 2020). In a hippocampus injury model, exogenous mitochondria altered metabolic pathways and inhibited the production of ROS, the growth of microglia and astrocytes, and the degeneration of neurons (Jia et al., 2023). In a cerebral ischemia-reperfusion injury model, exogenous mitochondrial transplantation increased cellular survival, relieved neurobehavioral deficits, decreased apoptosis levels, and infarct size. According to mitochondrial tracking, some of the exogenous mitochondria merged with the host cell’s mitochondria, while other sections were absorbed into lysosomes (Xie et al., 2021). Lin et al. transplanted fluorescently tagged soleus-derived allogeneic mitochondria into damaged spinal cords. The findings indicated that transplanted mitochondrial could still be observed in the spinal cord for up to 28 days and recovered the capacity for movement and perception, reduced the expression of dynamin-related protein 1 (DRP-1) as well as the level of demyelination, and reduced the cellular death and inflammatory reactions spurred on by spinal cord injury (Lin et al., 2022). Mitochondrial transplantation on the degeneration of oligodendrocytes could effectively restore overall mitochondrial activity in the ischemic brain. Myelin basic protein levels and morphologically intact axon numbers were higher in 21 days following injury and mitochondrial transplantation enhanced lipid synthesis and restored locomotion in the cortex (Chen et al., 2022). Isolated mitochondria are incorporated into the neuronal cells in traumatic brain injury (TBI) model. Mitochondrial transplantation preserved normal brain shape, decreased apoptosis, relieved astrogliosis, and microglia activation, and enhanced sensorimotor capabilities (Bamshad et al., 2023). After cardiac arrest, mitochondrial transplantation demonstrated that isolated mitochondria located together with mitochondria inside target neurons. Freshly extracted functioning mitochondria significantly enhanced 72-h survival in the in vivo studies and generated improvement in edema of the lungs, brain circulation, lactate and glucose levels, and neurological activity (Hayashida et al., 2023). Mitochondrial therapy may be employed in treating a variety of conditions, such as TBI, ischemic stroke, and neurodegenerative diseases.

The kidney is a crucial organ that is involved in several cellular processes, such as acid-base equilibrium and the regulation of electrolyte homeostasis (Bhargava and Schnellmann, 2017). Therefore, there is a large energy need for sustaining these essential processes. Acute kidney injury, nephrotoxicity, and chronic kidney injury are just a few of the disorders that can develop because of mitochondrial dysfunction in this vital organ. In a model of doxorubicin-induced nephrotoxicity, mitochondrial transplantation minimized cellular oxidative stress and enhanced tubular cell regeneration following renal injury. Additionally, mitochondrial transplantation decreased tubular cells’ accumulation of proteins, modified renal disorders, and diminished apoptosis (Kubat et al., 2021). In the renal ischemia-reperfusion injury model, mitochondrial transplantation improved the regeneration, proliferation, renal impairments, decreased DRP-1 fission protein dynamics, and reduced terminal deoxynucleotidyl transferase dUTP nick end labeling (TUNEL) and caspase-3 levels (Kubat et al., 2021). In the diabetic nephropathy model, isolated mitochondria were injected into the kidney and improved the morphology of renal cells (Konari et al., 2019). Mitochondrial transplantation attenuated blood parameters in the acute kidney injury model. The kidney with the transplanted mitochondria showed moderate acute tubular injury and decreased interleukin (IL-6) expression (Doulamis et al., 2020). Mitochondrial transplantation in vitro demonstrated increased proliferative capacity, ATP generation, and decreased ROS production in a renal transplantation model. Ex vivo studies revealed decreased kidney damage and RNAseq analysis found downregulation of genes such IL1A, CXCL8, and PIK3R1 that are involved in neutrophil recruitment as well as modification of genes most associated with mitochondrial biogenesis and energy metabolism (Rossi et al., 2023). As shown in preclinical studies, mitochondrial transplantation can improve the ability of renal cells to repair and proliferate while also reducing kidney damage and restoring renal function.

The liver is an essential organ with several activities, and because of this, it is extremely dependent on ATP and the functioning oxidative phosphorylation system (Chinnery and DiMauro, 2005). Hepatocytes that encounter excessive physiologic stress produce dysfunctional mitochondria. Mitochondrial transplantation restored the histological structure of the liver and diminished apoptosis, plasma alanine transaminase levels, and total oxidant in an acetaminophen-induced liver injury (Ulger et al., 2021). The mitochondria infiltrated hepatocytes by macropinocytosis in a carbon tetrachloride (CCl4)-induced liver injury model and cell survival recovered. Mitochondrial transplantation considerably improved liver function, reduced tissue fibrogenesis, activated antioxidant genes, and enhanced mitochondrial function via regulation of respiratory chain enzyme and mitophagy pathway (Zhao et al., 2021). The liver ischemia-reperfusion injury was alleviated by mitochondrial transplantation, which decreased blood alanine aminotransferase levels, necrosis of hepatocytes, an increase in TUNEL-positive cells, expression of cytochrome c, caspase 9, and 4-hydroxynonenal (Lin et al., 2013). Mitochondrial transplantation can protect liver cells from damage via restoration of mitochondrial stability.

It is well known that cardiovascular diseases are significantly influenced by mitochondrial dysfunction and the primary function of the myocardium is to provide sufficient contractile power to move blood throughout the body, which requires energy provided by the mitochondria (Pour et al., 2021). Mitochondrial transplantation attenuated cardiac blood parameters, infarct size, and apoptosis in myocardial ischemia-reperfusion injury (IRI). Serial echocardiograms revealed that after 10 min of reperfusion, mitochondrial transplantation returned to normal contraction (Masuzawa et al., 2013). When compared to untreated rats, hearts that received delayed mitochondrial transplantation following regional IRI demonstrated modified cardiac physiological functions. At the end of reperfusion, there was a variation in left ventricular (LV) functionality across groups. Importantly, mitochondrial transplantation dramatically reduced infarct size (Blitzer et al., 2020). Following 30 min of reperfusion injury, both single and serial injection mitochondrial transplantation dramatically improved coronary blood flow. Mitochondrial transplantation elevated ejection fraction developed pressure, improved regional function and strain analysis, and diminished infarct size in both mitochondria groups (Guariento et al., 2020). A polypeptide that permits transplanted mitochondria to be successfully received by cardiomyocytes may be beneficial with the treatment approach. The reduction of cellular death, macrophage infiltration, and the proinflammatory response due to mitochondrial transplantation through polypeptide was accomplished by improving the energetics and mechanical contraction of cardiomyocytes (Sun et al., 2023). In a doxorubicin-induced heart failure model, mitochondrial transplantation preserved cardiac function in vivo and prevented myocardial apoptosis. Also, it specifically enhanced the contraction of ventricular myocytes and the contractility of dystrophic cardiomyocytes (Zhang et al., 2023). Diabetes-related heart failure causes reduced mitochondrial function, which increases myocardial sensitivity to IRI. Although mitochondrial transplantation reduces IRI, its cardioprotective effects could be constrained when using diabetic mitochondria. Over time, mitochondrial transplantation led to a reduction in infarct size, which was correlated with the continuous activation of pathways. The effects of mitochondrial transplantation enhanced myocardial function. However, the cardioprotection provided by mitochondrial transplantation was less in diabetic recipients compared to nondiabetic recipients (Doulamis et al., 2022). Further studies might lead to the application of mitochondrial transplantation for subcellular biotherapy to prevent heart tissue damage and ultimately improve cardiac function.

Mitochondria have many different roles in skeletal muscle, and reducing their functionality can seriously harm muscle health (Kubat et al., 2023; Turkel et al., 2023). In an acute limb ischemia model, mitochondrial transplantation dramatically reduced infarct size and apoptosis in the skeletal muscles such as gastrocnemius, soleus. After reperfusion, DigiGait analysis revealed that stance time was reduced and stance factor was significantly increased by mitochondrial transplantation. Furthermore, there was no muscle injury after mitochondrial transplantation and a significant decrease in TUNEL-positive nuclei (Orfany et al., 2020). Mitochondrial transplantation did not affect on inflammation in the injured skeletal muscle model, and muscle mass and force declined in injured muscles seven days after injury, whereas collagen and other noncontractile tissue levels were much greater. The muscle wet weight and maximum muscular force returned to normal levels 14 days after injury due to mitochondrial transplantation (Alway et al., 2023). In the dexamethasone-induced atrophy model, mitochondrial transplantation enhanced muscle mass and lowered lactate concentration after 1 week in atrophic muscles and increased the muscle regeneration protein (desmin). An important finding was that the AMP-activated protein kinase (AMPK)-mediated Akt-forkhead box transcription factors (FoxO) signaling pathway significantly decreased the muscle-specific ubiquitin E3-ligases Muscle RING finger 1 (MuRF1) and muscle atrophy F-box (MAFbx)/atrogin-1after mitochondrial transplantation (Kim et al., 2023). Osteoarthritis (OA) progression is at risk due to chondrocyte mitochondrial dysfunction. In OA animals with mitochondrial transplants, there was a reduction in pain, cartilage degradation, bone loss, and mitochondrial transplantation significantly reduced the transcript levels of inflammation (IL-1, tumor necrosis factor-alpha (TNF-)), chemokines (matrix metallopeptidase 13, monocyte chemoattractant protein-1 (MCP-1)) (Lee et al., 2022). Because of its advantages such as decreasing oxidative stress, and increasing regeneration, mitochondrial transplantation could serve as one of the future therapy strategies for skeletal muscle problems.

Atherosclerotic lesions may develop if extra cholesterol is not eliminated as foam cells will accumulate. Healthy mitochondria delivered into macrophages with elevated cholesterol levels promote phagocytosis and prevent a shift toward foam cells. The process of their phagocytic machinery was enhanced by mitochondrial transplantation, which also restored phagocytosis and reduced lipid deposition (Játiva et al., 2022). Retinal pigmented epithelium cells have a high mitochondrial capacity. Isolated mitochondria were injected into the retinal spaces of rats that had hereditary retinal degeneration. A histological examination found that mitochondrial engraftment significantly decreased retinal layer degeneration in rats and the retinal electrical signalling increased after mitochondrial transplantation (Wu et al., 2022). The efficacy of male and female mitochondrial transplantation was investigated in a malignant melanoma model. The findings revealed mitochondrial transplantation effectively prevented tumor formation and lung migration, cell cycle arrest and generation of cell death, and tumor cell proliferation in vitro. Following mitochondrial transplantation on metastatic melanoma, transcriptome analysis demonstrated that overall chromosomal silencing was strongly associated with the mitochondria’s ability to eradicate cancer. Furthermore, compared to male mitochondria, female mitochondria indicated more robust antitumor activity, and female mitochondria were probably more likely to produce better mitochondria-nuclear interaction (Yu et al., 2021). In an acute lung injury model, mitochondrial transplantation significantly increased arterial oxygen contents and diminished co_2_ tension, promoted ATP concentrations, and preserved alveolar-capillary barrier function. Furthermore, after mitochondrial transplantation, the infiltration of inflammatory cells into lung tissue was reduced, and the generation of endothelial nitric oxide synthase (eNOS) and relaxation responses to acetylcholine were also enhanced in injured pulmonary arteries (Pang et al., 2022). A variety of models have been applied in vivo mitochondrial transplantation applications, and these models have the potential to serve in the clinic in the future.

## 5. Clinical trials of mitochondrial transplantation

As previously explained, numerous preclinical studies regarding mitochondria transplantation have been performed, but only two clinical trials have recently been finalized and these clinical trials are promising. However, some clinical studies are still ongoing.

Pediatric patients with ischemia-reperfusion damage were included in the first clinical trial on humans (Emani et al., 2017). Four of the five patients who received mitochondrial transplantation were successfully weaned from extracorporeal membrane oxygenation (ECMO), however, eventually only three of them lived. Due to mitochondrial transplantation, these patients do not have any inflammation or rejection. The second clinical trial included 24 patients, including 14 controls and 10 mitochondria transplantation (Guariento et al., 2021). Only 29% of patients who did not receive mitochondrial therapy were able to discontinue using an ECMO machine after a week, compared to 80% of patients who received treatment. Prospective clinical studies need to be conducted to evaluate the therapy’s safety, effectiveness, and ideal dosage.

Ongoing clinical studies are “Transplantation of Autologously Derived Mitochondria Following Ischemia (NCT02851758)” and “Autologous Mitochondrial Transplant for Cerebral Ischemia (NCT04998357)”.

## 6. The challenges of mitochondrial transplantation

There has been numerous research on mitochondrial transfer/transplantation, however, there are still problems that need to be explored. These challenges are an immune response, the calcium concentration, the quality and efficiency of isolated mitochondria, and the application route of transplantation.

The immune response to mitochondrial damage-associated molecular patterns (DAMPs) may be triggered by the transplanted mitochondria, but the inflammatory response is probably complex. While some studies have reported an immunological response, others have found none. After intraperitoneal injection of syngeneic and allogenic mitochondria, McCully et al. found that there was no significant elevation in immunological markers (Ramirez-Barbieri et al., 2019). Lin et al. reported that the production of cytokines and chemokines that promote inflammation was found to be enhanced by interactions with mitochondria (Lin et al., 2019).

It is generally known that calcium ions harm mitochondria, triggering hole development and membrane degradation. Bertero et al. expressed that an elevated plasma calcium ion concentration promotes mitochondrial survival improbable and reported that mitochondria are severely damaged when there is calcium available (Bertero et al., 2018; Bertero et al., 2020). Studies demonstrated that transplanted mitochondria, however, were incorporated and effectively incorporated into target cells in recipient cells with a calcium environment (Kesner et al., 2016; Gollihue et al., 2018).

Another challenge is that the integrity and functionality of mitochondria are impacted by isolation methods (Kubat et al., 2021). We do not yet know how many mitochondria are active and functioning within the cytosol. Multiple analyses may be used to examine the quality and efficiency of isolated mitochondria, and these analyses are performed in a small sample group in mitochondrial transplantation studies. After isolated mitochondria can stay active on ice for about an hour, storage has a substantial impact on transplantation efficacy (McCully et al., 2017). As a result, it is essential to develop an effective method for maintaining mitochondria’s stability and bioenergetic activity.

Numerous studies have demonstrated various methods of delivery of mitochondria, including direct injection, systemic injection, intranasal administration, and coincubation (Bobkova et al., 2021; Kubat et al., 2021). Mitochondrial infusion directly to tissues has prompted several animal and even human studies (Masuzawa et al., 2013; Emani et al., 2017). In comparison to direct injection, systemic administration demands a significantly higher dose of mitochondria and requires a lot longer to deliver mitochondria to a target cell (Ulger and Kubat, 2022).

## 7. Conclusion

This review describes the current status of mitochondrial transplantation studies. Applications for in vitro and in vivo mitochondrial transfer/transplantation are discussed in this review, and they serve as a framework of theory for the investigation of natural mitochondrial transfer. Although the processes behind mitochondrial transfer and transplantation remain little known, these applications still represent therapeutic potential. The immunological response, ideal dosage and delivery technique, viability, activity, and preservation of isolated mitochondria, however, should be standardized in mitochondria transplantation with wide international collaboration.

## Figures and Tables

**Figure:**
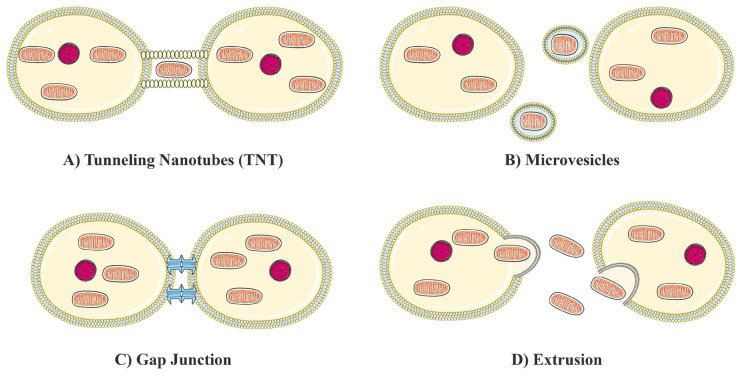
Natural Mitochondrial Transfer. A) Tunneling Nanotubes (TNT), B) Extracellular vesicles, C) Gap Junction, D) Extrusion. The schematic art pieces used to produce the figure in this article were provided by Servier Medical art (http://servier.com/Powerpoint-image-bank). Servier Medical Art by Servier is licensed under a Creative Commons Attribution 3.0 Unported License.

**Table:** Summary of artificial mitochondrial transplantation and transfer: In vivo applications.

Models	Result	Reference
Acute ischemia stroke model	Decreased apoptosis, microglial activation process, infarction size, and improved motor functioning	Pourmohammadi-Bejarpasi, et al. 2020
Hippocampal injury model	Prevented ROS, the proliferation of microglia and astrocytes	Jia, et al. 2023
Cerebral ischemia-reperfusion injury model	Improved cellular survival, lowered ROS, apoptosis infarct size, alleviated neurobehavioral impairments	Xie, et al. 2021
Spinal cord injury model	Restored movement and sense, reduced the expression of DRP-1, demyelination, cellular death and inflammatory reactions	Lin, et al. 2022
Ischemic brain model	Prevented degeneration of oligodendrocytes, increased myelin basic protein and intact axon, olig2, lipid synthesis signaling and restored locomotion	Chen, et al. 2022
Traumatic brain injury model	Preserved brain morphology, decreased apoptosis, astrogliosis and microglia activation, and enhanced sensorimotor ability	Bamshad, et al. 2023
Neuron damage after cardiac arrest model	Reduced lung edema, enhanced brain microcirculation, lactate, glucose levels, and neurological function	Hayashida, et al. 2023
Doxorubicin-induced nephrotoxicity model	Reduced cellular oxidative stress, tubular protein accumulation, Caspase3 levels and increased tubular cell regeneration, Bcl-2 levels	Kubat, et al. 2021
Renal ischemia-reperfusion injury model	Increased tubular proliferation and regeneration, improved renal impairments, decreased DRP-1, TUNEL and Caspase-3 levels	Kubat, et al. 2021
Diabetic nephropathy model	Improved the cellular morphology and the structure of renal cells	Konari, et al. 2019
Acute renal damage model	Prevented acute tubular injury and reduced IL-6 expression	Doulamis, et al. 2020
Renal transplantation model	Enhanced proliferation and ATP generation, reduced ROS, renal injury, modified genes of mitochondrial biogenesis, neutrophil recruitment, energy metabolism	Rossi, et al. 2023
Acetaminophen-induced liver injury model	Restored liver morphology and diminished apoptosis, alanine transaminase and oxidant levels	Ulger, et al. 2021
CCl4-induced liver damage model	Restored cell viability, improved liver function, reduced tissue fibrogenesis, activated antioxidant genes, and enhanced mitochondrial function	Zhao, et al. 2021
Liver ischemia-reperfusion model	Reduced alanine transaminase, necrosis of hepatocytes, TUNEL positive cells, apoptotic markers	Lin, et al. 2013
Heart ischemia-reperfusion injury model.	Attenuated creatine kinase MB, cardiac troponin-I, apoptosis, and infarct size	Masuzawa, et al. 2013
Heart ischemia-reperfusion injury model	Decreased myocardial infarctions and improved the efficiency of myocardium	Blitzer, et al. 2020
Heart ischemia-reperfusion injury model	Improved coronary blood flow, regional function and strain analysis, elevated ejection and diminished infarct size	Guariento, et al. 2020
Heart ischemia-reperfusion injury model	Increased cardiomyocyte vitality, decreased apoptosis, and proinflammatory activity	Sun, et al. 2023
Doxorubicin-induced heart failure model	Improved cardiac function, prevented myocardial apoptosis, increased contraction in ventricular myocytes, contractility in dystrophic cardiomyocytes	Zhang, et al. 2023
Diabetes-related heart failure model	Decreased infarct size, enhanced myocardial function and reduced cardioprotection in diabetic recipients compared to nondiabetic recipients	Doulamis, et al. 2022
Acute limb ischemia model	Reduced size of infarction, apoptosis, and inflammation increased hindlimb efficiency and ATP content	Orfany et al., 2020
Muscle injury model	Repaired muscle fibers, enhanced regeneration and muscular function	Always et al., 2023
Dex-induced skeletal muscle atrophy	Lowered lactate concentration, increased muscle regeneration, decreased ubiquitin E3-ligases MAFbx and MuRF-1	Kim, et al. 2023
Osteoarthritis model	Reduced pain, cartilage degradation, bone loss, inflammation	Lee, et al. 2022
Cholesterol-loaded macrophages model	Promoted phagocytosis, reduced lipid deposition	Játiva, et al. 2022
Retina degeneration model	Decreased retinal degeneration, increased retinal electrical impulse	Wu, et al. 2022
Malignant melanoma model	Prevented tumor formation and lung migration, cell cycle arrest and generation of cell death, female mitochondria indicated more robust antitumor activity	Yu, et al. 2021
Endotoxin-induced acute lung injury model	Increased arterial O_2_ contents, diminished CO_2_ tension, promoted ATP concentrations, and preserved alveolar-capillary barrier function, reduced infiltration of inflammatory cells and increased eNOS and relaxation responses to acetylcholine	Pang, et al. 2022

Reactive oxygen species (ROS), dynamin-related protein 1 (DRP-1), terminal deoxynucleotidyl transferase dUTP nick end labeling (TUNEL), interleukin 6 (IL-6), Muscle RING Finger-1 (MuRF1), tumor necrosis factor-alpha (TNF-α), endothelial nitric oxide synthase (eNOS)
